# Allergic rhinitis: the “Ghost Diagnosis” in patients with asthma

**DOI:** 10.1186/s40733-015-0008-0

**Published:** 2015-09-07

**Authors:** Maureen Egan, Supinda Bunyavanich

**Affiliations:** 1 Division of Allergy and Immunology, Department of Pediatrics, Icahn School of Medicine at Mount Sinai, One Gustave L. Levy Place #1198, New York, NY 10029 USA; 2 Department of Genetics and Genomic Sciences, and Mindich Child Health and Development Institute, Icahn School of Medicine at Mount Sinai, New York, NY USA

**Keywords:** Allergic rhinitis, Asthma, Allergen sensitization, Allergen immunotherapy

## Abstract

Allergic rhinitis (AR) is a common comorbidity of asthma that contributes to asthma severity. Although over 80 % of asthmatics have AR, the condition is frequently underdiagnosed in subjects with asthma. AR itself is also a highly prevalent condition, affecting 10-30 % of adults and up to 40 % of children. AR has been associated with both increased risk of asthma development and asthma severity. The exact mechanisms underlying these relationships have yet to be fully elucidated, but evidence supports a role for allergen sensitization. Compared to those with asthma alone, patients with comorbid AR and asthma have greater use of health care resources, including visits to the general practitioner, emergency department and hospitalizations. Pharmacological treatment of AR reduces this health care burden. Immunotherapy for AR improves both asthma and rhinitis symptoms in addition to preventing future allergen sensitizations and asthma development. Appropriate recognition, diagnosis and treatment of AR can significantly reduce asthma morbidity and improve quality of life.

## Introduction

Allergic rhinitis (or hayfever) is a common comorbidity of asthma that contributes to asthma severity [1, 2]. Although over 80 % of asthmatics have comorbid allergic rhinitis (AR) [3], the condition is frequently underdiagnosed in individuals with asthma [4, 5]. Treatment of comorbid AR can reduce the odds of asthma-related healthcare, specifically emergency room visits and hospitalizations, by up to 80 % [6]. Appropriate recognition, diagnosis and treatment of AR therefore represent paths toward improving disease management and quality of life for asthmatics. In this article, we address the “ghost diagnosis” of AR in subjects with asthma by discussing the clinical presentation of AR, evidence for AR as a risk factor in asthma, mechanisms underlying AR comorbidity with asthma, and clinical implications for its diagnosis and management in asthmatics.

## Review

### Clinical presentation and underdiagnosis of allergic rhinitis

AR is one of the most common chronic diseases world-wide, affecting 10–30 % of adults and up to 40 % of children [7]. Its global prevalence continues to increase [7], with over 500 million individuals affected worldwide [3]. The prevalence of AR is increasing in countries with previously low prevalence while plateauing in areas of highest prevalence [3]. In the United States, physician-diagnosed AR affects approximately 15–40 % of the population [8, 9]. Both the Global Strategy for Asthma Management and Prevention and National Asthma Education and Prevention Program’s Expert Panel Report (EPR-3) guidelines for the diagnosis and management of asthma recommend an evaluation for comorbid AR in patients with asthma [10, 11].

AR is an IgE-mediated disease characterized by one or more symptoms including nasal congestion, rhinorrhea, sneezing and itching on consecutive days [7, 12]. According to Joint Task Force guidelines set forth by the American Academy of Allergy, Asthma and Immunology (AAAAI), American College of Allergy, Asthma and Immunology (ACAAI) and the Joint Council on Allergy, Asthma and Immunology, AR can be classified as seasonal, perennial and episodic [7]. Perennial AR is typically caused by sensitization to indoor allergens such as dust mites, mold and animal dander, while seasonal AR is most often due to sensitization to pollen allergens [13]. Episodic AR results from sporadic exposures to aeroallergens that are not typically encountered, such as visiting a farm or home with animal allergens that an individual would not typically encounter [7].

Alternatively, the international working group Allergic Rhinitis and its Impact on Asthma (ARIA) suggests categorizing AR as intermittent or persistent. This is motivated by the fact that aeroallergens may be present seasonally in one area and year-round in other areas [13]. In the ARIA classification system, intermittent refers to symptoms occurring less than 4 days a week or for less than 4 consecutive weeks, while persistent refers to symptoms present more than 4 days per week and for more than 4 consecutive weeks. The ARIA working group additionally classifies severity of AR as mild and moderate/severe. Patients have mild AR if they do not have any of the following characteristics: sleep disturbance, impairment of daily activities, impairment of school/work and symptoms. Patients with one or more of the above characteristics are considered to have moderate/severe AR [3, 13].

The initial evaluation of AR includes history and physical examination. The history should include careful attention to environmental exposures with a focus on precipitating factors and quality of life assessment [7]. Physical exam findings may include rhinorrhea, enlargement and pallor of the inferior nasal turbinates, conjunctival injection and increased lacrimation, Dennie-Morgan lines, allergic shiners, nasal crease, and sinus tenderness [14]. Further testing for allergen-specific IgE antibodies should be done to assess for sensitization to suspected allergens [7]. Although allergen skin prick testing is preferred, in vitro assays for serum allergen-specific IgE can be performed for patients who cannot have skin testing performed due to dermatographism or recent oral antihistamine use [7]. The sensitivity of specific IgE in vitro assays compared with skin prick tests is approximately 70–75 % [7].

AR is underdiagnosed, as its symptoms may not always lead to significant quality of life impairment [3]. A study of allergic rhinitis symptoms in a diverse US urban population of 166 children with known asthma identified rhinitis symptoms in 72 % of subjects, with AR undiagnosed in 53 % [15]. A cross-sectional population-based study in six Colombian cities noted that the prevalence of subjects reporting current AR symptoms was 32 %, while the prevalence of physician diagnosed AR was lower at 14 % [16]. A separate study of Costa Rican children with asthma showed that AR (as defined by characteristic symptoms and allergen skin test reactivity) had a prevalence of 80 %, while physicians diagnosed AR in only 27 % of the cohort [4]. A physician’s diagnosis was therefore only 29.5 % sensitive for skin-test-reaction positive AR [4]. A recent study in Puerto Rican children additionally reported that physicians correctly diagnosed AR in only 15.3 % of children with asthma and 3.5 % of children without asthma [5].

Underdiagnosis of AR also occurs in adult populations. Nolte et al. evaluated subjects age 14–44 years in Denmark and found that allergic rhinitis was underdiagnosed in 32 % of subjects [17]. Similarly, a telephone survey of European individuals >18 years of age noted that among patients who reported signs and symptoms consistent with allergic rhinitis, 45 % had never been diagnosed by a physician [18].

Evaluation of AR by an allergist/immunologist improves patient health-related quality of life outcomes including general health, social functioning and vitality [19]. Harmsen et al. found that patients with concurrent asthma and AR who received care by a specialist had improvements in both their asthma and rhinitis quality of life as assessed by standardized questionnaires (AQLQ-Asthma Quality of Life Questionnaire and RQLQ-Rhinitis Quality of Life Questionnaire). With regard to asthma control specifically, 67 % of patients followed by a generalist during the three year study time period had unchanged or worsened asthma control compared to 45 % of patients followed by a specialist (P value <0.01) [20]. Referral to an allergist/immunologist is currently recommended for many clinical circumstances including, but not limited to, inadequately controlled AR symptoms, reduced quality of life, determination of allergen sensitizations, and/or in patients with comorbidities such as asthma [7].

### Allergic rhinitis as a risk factor for asthma

AR and asthma have high comorbidity [13]. Over 80 % of asthmatics have AR [3] while 10–40 % of individuals with AR have asthma [13, 21]. AR is a risk factor for asthma [4, 5, 22, 23], and the diagnosis of AR can precede asthma [1, 24]. Studies of both adult [25, 26] and pediatric populations [24] provide evidence for increased risk of asthma development in individuals with AR. Burgess et al. found that childhood AR was associated with a 7-fold increased risk of asthma in preadolescence (HR 7.12, 95 % CI 3.97-12.75) and an over 4-fold increased risk of asthma in adolescence (HR 4.34, 95 % CI 2.23-8.46). Furthermore, childhood AR increased the likelihood of having asthma that persisted into middle age by 3-fold (95 % CI 1.98-4.56) [27]. Similarly, a prospective 8-year study of children with a history of recurrent wheezing demonstrated that a history of AR was associated with significantly increased odds of persistent asthma symptoms (OR 15.8, 95 % CI 6.1-40.8) [28].

Among subjects with AR, subjects with more severe AR symptoms are more likely to suffer from asthma, and potentially more severe asthma, than subjects without AR or those with milder AR [29]. For example, the DREAMS study group found that 33 % of subjects with moderate/severe persistent rhinitis had asthma compared to 2 % of the control population [30]. Similarly, Ponte et al. demonstrated that the severity of AR was positively correlated with asthma severity, as evidenced by a 3.8 fold increase in the odds of emergency room visits (95 % CI 2.00-7.35) in patients with moderate to severe rhinitis as compared to patients without rhinitis. Additionally, patients with moderate to severe rhinitis had a 12.7 fold increase (95 % CI 1.73-92.85) in the odds of having uncontrolled asthma compared to those without rhinitis [31]. Sasaki et al. noted that among pediatric patients with asthma, uncontrolled asthma was present in 9.2 % of those without rhinitis, 15.3 % of those with mild-moderate rhinitis, and 29.2 % of those with severe rhinitis [32]. The ARIA working group has proposed that AR and asthma may be manifestations of one syndrome in two parts of the respiratory tract, with more severe AR corresponding directly with more severe asthma [3].

A minority of studies do not support an association between AR severity and asthma status. A cross-sectional study in Italy found asthma prevalence to be independent of AR severity classified by the ARIA system [33]. The same authors found weak associations between AR class and asthma therapies [34]. The group did, however, find a significant association between severity of AR and medications for AR treatment [34].

In sum, the bulk of the evidence supports that AR status and severity are associated with asthma. The majority of studies reaffirm current guidelines set by the Joint Task Force recommending that pulmonary functions tests be considered in patients with AR given the high risk of comorbid asthma [7].

### Mechanism of comorbidity: allergen sensitization

The precise mechanisms underlying comorbid asthma and AR have yet to be fully elucidated. The physical connection between the nasal passages, paranasal sinuses, and lower respiratory tract provide the basis for the ‘one airway, one disease’ theory [35]. AR is an IgE mediated disease leading to respiratory tract inflammation in response to environmental allergens to which one is sensitized [3]. It is hypothesized that IgE fixes to membranes of mast cells, and subsequent mast cell accumulation in the united airway mucosa contributes to both AR and asthma [3]. Additionally, mucosal inflammation as well as physical stimuli can trigger nasal neurogenic reflexes via sensory, parasympathetic and sympathetic pathways that produce symptoms such as pruritus and sneezing [3].

The prevalence of sensitization to inhalant allergens is reported to be greater than 40 % in the United States and Europe [12, 36]. Allergen sensitization is an important risk factor in the development of all atopic disease, including AR and asthma [36]. The German Multi-center Allergy Study demonstrated that mere exposure to inhalant allergens was not a risk factor for future asthma [37]. However, *sensitization* to inhalant allergens and persistence of this sensitization were both associated with wheezing at ages 3–7 years old, with wheezing observed in ~30 % of dust mite-sensitized children compared to only 10 % in those not sensitized (*p* < 0.001) [37]. A follow up evaluation of the cohort showed that 90 % of subjects with non-allergic wheezing progressed towards normal lung function at puberty, compared to 56.2 % for atopic wheezers (*P* = 0.0002) [38]. The study further demonstrated that early sensitization, particularly to perennial allergens, was associated with increased risk of wheezing. Specifically, sensitization to perennial allergens before age 3 years was associated with a 15.5 increased odds (95 % CI 3.32-72.42) of wheezing at school age (ages 5–7 years old) while subjects with perennial sensitization at 7 years old had a 5.7 increased odds (95 % CI 2.38-23.70) of wheezing at school age [38]. Seasonal sensitization was similarly associated with an increased risk of wheezing, but to a lesser degree [38].

Similarly, in a high risk Canadian birth cohort, subjects who were sensitized to indoor allergens such as dust mite, cat and dog were 3–4 times more likely to have asthma at age seven compared to those without evidence of sensitization (sensitization to house dust mite OR 4.81, 95 % CI 2.47-9.34; sensitization to dog OR 3.81, 95 % CI 1.79-9.22; sensitization to cat OR 3.33, 95 % CI 1.72-6.56) [39]. In the Childhood Origins of Asthma (COAST) high birth risk cohort, poly-sensitization to aeroallergens at age 6 was associated with a 3.4-fold increased risk (95 % CI 1.7-6.7) of asthma [40]. Thus, allergen sensitization likely plays a central role in subjects with AR and asthma.

In addition to asthma status, allergen sensitization has also been associated with asthma severity [41]. The German Multicenter Allergy Study demonstrated that atopic wheezers at school age had increased severity of asthma symptoms, with 23.7 % of subjects with atopic wheezing experiencing more than 4 episodes of wheezing in one year compared to 8.9 % in nonatopic wheezers (*P* = 0.0238) [38]. A separate study of school-aged children with asthma reported higher rates of sensitization to aeroallergens in children with severe asthma compared to those with mild to moderate asthma. All subjects with severe asthma were atopic (as defined by an elevated sIgE or more than one positive skin prick reaction) while 76 % of children with mild-to-moderate asthma were atopic (*P* = 0.12) [42]. More specifically, children with severe asthma had higher numbers of positive skin prick reactions to aeroallergens, with a mean of 5 positive skin tests in the severe group and 3 in the mild-to-moderate group (*P* = 0.009) [42]. However, it should be noted that not all studies have demonstrated an association between asthma severity and allergen sensitization [43].

Allergen sensitization may have direct effects on the respiratory tract [44]. Approximately 23 % of subjects with AR demonstrate increased bronchial reactivity to methacholine, and subjects with perennial AR symptoms exhibit greater bronchial reactivity than those with seasonal symptoms [45, 46, 47]. Subjects with seasonal AR also demonstrate seasonal increases in bronchial hyperresponsiveness, with mean methacholine PD20 (dose of methacholine causing a 20 % fall of FEV1) of 352 μg/ml during the pollen season compared to 448 μg/ml outside the pollen season (*p* < 0.05) [48]. Despite these results, it remains unclear whether AR represents an earlier clinical manifestation of disease in atopic individuals who subsequently develop asthma, or if AR and sensitization are themselves causal factors to asthma [3].

### Clinical implications of comorbid AR in subjects with asthma

Subjects with comorbid asthma and AR experience greater asthma severity and health care utilization than asthmatic subjects without AR [1, 49]. Among children with asthma, comorbid AR is associated with a 2.3-fold increase in risk (95 % CI 1.42-3.91) for hospitalization for asthma and a 29 % increase in general practitioner visits for asthma (*p* < 0.0001) [1]. Similarly, in subjects age 15–72 years with asthma, self-reported concurrent AR symptoms was associated with a 1.35 increased risk of an asthma attack (95 % CI 1.03-1.77) and a 2.35 increased risk of an emergency department visit (95 % CI 1.12-4.8) compared to asthmatic subjects without AR symptoms during a one year study period [29]. Price et al. demonstrated that adults with concomitant AR and asthma had more annual visits to their general practitioner, with a mean increase of 0.42 visits per patient (95 % CI 0.42-0.43) and a 1.5 increased odds of being hospitalization (95 % CI 1.03-2.24) compared to adults with asthma alone [50]. Similarly, Halpern et al. demonstrated that patients with asthma and AR received 50 % more prescription for asthma-related medications annually than those with asthma only [51].

Comorbid AR can provide insight into asthma prognosis. The Norwegian Environment and Child Asthma (ECA) study demonstrated that the severity of airway obstruction at age 2 years and specific IgE levels to inhalant allergens could predict asthma at age 10 years and explain up to 30 % of the asthma variance observed [52]. The ECA study additionally demonstrated that males with an elevated serum IgE to inhalant allergens at age 2 years had a 1.58 increased odds (95 % CI 1.26-2.00) of having asthma at age 10 years; a similar relationship was not observed in girls [52]. Similarly, the commonly used Asthma Predictive Index (API), which estimates the persistence of wheezing at school age based on factors in the first 3 years of life, includes physician-diagnosed AR as a minor criterion [53]. However, the National Institutes of Allergy and Infectious Diseases, National Heart Lung Blood Institute, and Mechanisms of the Development of Allergy program recently reviewed over 130 birth cohort studies in asthma and allergic diseases at a workshop and concluded that the interplay between asthma, AR and atopic dermatitis still has many unanswered questions and the natural history of these conditions cannot definitively be predicted [54].

### Treatment of AR in subjects with asthma

Pharmacologic treatment of comorbid AR in asthmatic patients is essential, as treatment of concomitant AR reduces health care utilization [6, 55]. In a retrospective cohort study of subjects with AR and asthma, Crystal-Peters et al. found that the risk of asthma-related events, which included hospitalizations and emergency department visits, in the group treated for AR was 1.3 % compared to 6.6 % in the untreated group. An incidence density ratio indicated that the risk of an asthma-related event in the treated group was approximately half that in the untreated group (IDR 0.49, *P* = 0.001) [55]. Similarly, Adams et al. demonstrated that children older than 5 years with AR and asthma who had received an intranasal steroid had a 0.7 relative risk of emergency department visits (95 % CI 0.59-0.94) compared to those not treated [56]. It should be noted that these studies were observational and subject to potential bias including possible differences in quality of care [57].

Guidelines that describe treatment of AR have been published by the Joint Task Force in the US [7], ARIA working group [3] and as part of clinical references [14]. To briefly summarize, current pharmacologic treatment of AR focuses on intranasal corticosteroids, oral anti histamines (with a preference towards later generation products that are less sedating), leukotriene receptor antagonists, nasal antihistamines, and ocular agents [3]. Intranasal glucocorticoids are thought to be the most effective pharmacotherapy for seasonal AR [12].

For patients with concomitant AR and asthma, leukotriene receptor antagonists (LTRA) in particular can be effective when used in patients older than 6 years [3]. Montelukast (a LTRA) has been found to improve nasal and bronchial symptoms with reduction of beta agonist use in subjects with comorbid seasonal AR and asthma [58, 59]. Fewer trials have been done in younger children, but there is evidence that montelukast may be beneficial in this population, particularly in patients who intermittently develop symptoms after upper respiratory tract infections [60].

Reduction of allergen exposure represents an intuitive approach for AR management in subjects with asthma. However, single preventative measures in subjects with dust mite sensitization, AR and asthma do not appear to be effective [61]. A recent Cochrane review examining randomized trials of subjects with asthma who underwent chemical or physical methods to reduce dust mite allergen exposure concluded that there was no difference in asthma symptoms or medications scores with allergen exposure reduction [62]. However, this Cochrane review included studies that did not effectively decrease allergen exposure, and many interventions were of short duration [63]. The EPR-3 guidelines recommend a multifaceted approach for patient education regarding environmental control and allergen avoidance in patients with asthma, as single interventions are often ineffective [11].

Despite current pharmacologic options and allergen avoidance options, approximately 1 out of 3 children and 2 out of 3 adults report poor relief of AR symptoms. For this subpopulation, allergen immunotherapy may be considered [8, 64]. Allergen immunotherapy includes both sublingual immunotherapy (SLIT) and subcutaneous immunotherapy (SCIT). The FDA recently approved SLIT for use in the United States [65]. Given this recent approval in the US, differences exist between European-based and US-based guidelines for immunotherapy treatment of AR [66].

Immunotherapy leads to improvement in asthma symptoms when used in subjects with concurrent AR [67]. Both SCIT and SLIT for AR reduces asthma symptoms and asthma medication use [68, 69]. A recent Cochrane review which examined SCIT in relation to asthma care noted that it was associated with a significant improvement in asthma symptoms. The review concluded that treating three patients with SCIT would avoid one episode of deterioration in asthma symptoms, and treating four patients with SCIT would avoid one patient requiring an increase in medication [70]. Similarly, another review examining the effect of SLIT on asthma reported that eight of the 13 studies which examined this association reported greater than 40 % improvement in asthma symptoms [69]. Regarding SCIT vs SLIT, the best route of immunotherapy delivery has yet to be established, but limited evidence thus far supports SCIT over SLIT for reduction of asthma and AR symptoms [71].

Immunotherapy for AR may prevent new allergen sensitizations [72–74] and prevent the development of asthma [75]. In children with pollen allergy, immunotherapy has a preventive effect on the development of future asthma. The Preventative Allergy Treatment study demonstrated that subjects treated with immunotherapy had a 2.68 reduced risk (95 % CI 1.3-5.7) of having asthma during the 5 year follow up [76, 77]. Similarly, a randomized trial of SLIT for grass pollen allergy reported that subjects who were not treated developed asthma 3.8 times more frequently (95 % CI 1.5-10.0) over the following 3 years than those treated [78]. These studies support current guidelines that recommend consideration of immunotherapy for patients with AR and mild/moderate persistent asthma [11].

## Conclusions

Most individuals with asthma have AR. AR is associated with the development and severity of asthma. It is likely that treatment of AR with medications or allergen immunotherapy can significantly reduce asthma morbidity. Current guidelines such as those from GINA and EPR-3 recommend an evaluation for AR in all patients with asthma. A large body of evidence supports such guidelines, as the recognition, diagnosis, and treatment of AR in subjects with asthma can reduce asthma morbidity and improve quality of life.

## Abbreviations

AR: Allergic rhinitis
IgE: immunoglobulin E
LTRA: Leukotriene receptor antagonists
SLIT: Sublingual immunotherapy
SCIT: Subcutaneous immunotherapy
